# Glioma-Stem-Cell-Derived Exosomes Remodeled Glioma-Associated Macrophage via NEAT1/miR-125a/STAT3 Pathway

**DOI:** 10.3390/cancers16142500

**Published:** 2024-07-09

**Authors:** Tong Pan, Dong-Kun Xie, Juan Li, Yu-Jie Qiang, Song-Yuan Fan, Ting-Ting Wang, Yuan-Yuan Han, Jian Zang, Yang Yang, Jun-Long Zhao, San-Zhong Li, Shuang Wu

**Affiliations:** 1 Department of Neurosurgery, Xijing Hospital, Air Force Medical University, Xi’an 710032, China; zhang121309@163.com (T.P.); juanli834@126.com (J.L.); yujieqiang106@163.com (Y.-J.Q.); wtt11024@126.com (T.-T.W.); yuayuanhan1111@163.com (Y.-Y.H.); 2 Department of Biochemistry and Molecular Biology, Air Force Medical University, Xi’an 710032, China; 3 Key Laboratory of Resource Biology and Biotechnology in Western China, Ministry of Education, Faculty of Life Sciences, Northwest University, Xi’an 710069, China; xdk19971103@163.com (D.-K.X.); yang200214yy@nwu.edu.cn (Y.Y.); 4 State Key Laboratory of Cancer Biology, Department of Medical Genetics and Developmental Biology, Air Force Medical University, Xi’an 710032, China; bio_junlongzhao@163.com; 5 Department of Neurosurgery, The Air Force Hospital of Central Theater of PLA, Datong 037000, China; fsy7702024@163.com; 6 Department of Radiotherapy, Xijing Hospital, Air Force Medical University, Xi’an 710032, China; jianzangbio@163.com

**Keywords:** glioblastoma (GBM), exosome, stem cell, NEAT1, STAT3, miR-125a

## Abstract

**Simple Summary:**

Glioblastomas (GBMs) are considered the most lethal cancer in the central nervous system (CNS), whose malignant phenotypes are majorly attributed to glioma stem cells (GSCs). Despite combined surgical radiotherapy with temozolomide chemotherapy and tumor-treating fields (TTFs), the tumor almost always recurs near the resection site. Besides the contribution of GSCs, the tumor microenvironment (TME) also plays an important role in glioma recurrence. Our work has demonstrated that GSC-derived exosomes carry lncRNA NEAT1 to promote the M2 polarization of glioma-associated macrophages (GAMs). Further mechanism exploration indicated that NEAT1 represses the expression of miR-125a in GAMs significantly. The decrease in miR-125a induces the elevation of target gene STAT3, which is required for macrophage M2 polarization. The development of M2-like GAMs contributes to the immunosuppressive microenvironment and glioma progression. Our findings elucidate the functions and mechanisms of the crosstalk between GSCs and GAMs via exosomes, providing new therapeutic targets and strategies for glioma.

**Abstract:**

Glioblastoma (GBM), as the most common primary brain tumor, usually results in an extremely poor prognosis, in which glioma stem cells (GSCs) and their immunosuppressive microenvironment prominently intervene in the resistance to radiotherapy and chemotherapy that directly leads to tumor recurrence and shortened survival time. The specific mechanism through which exosomes generated from GSCs support the creation of an immunosuppressive microenvironment remains unknown, while it is acknowledged to be engaged in intercellular communication and the regulation of the glioma immunosuppressive microenvironment. The elevated expression of LncRNA-NEAT1 was found in glioma cells after radiotherapy, chemotherapy, and DNA damage stimulation, and NEAT1 could promote the malignant biological activities of GSCs. Emerging evidence suggests that lncRNAs may reply to external stimuli or DNA damage by playing a role in modulating different aspects of tumor biology. Our study demonstrated a promotive role of the carried NEAT1 by GSC-derived exosomes in the polarization of M2-like macrophages. Further experiments demonstrated the mediative role of miR-125a and its target gene STAT3 in NEAT1-induced polarization of M2-like macrophages that promote glioma progression. Our findings elucidate the mechanism by which GSCs influence the polarization of M2-like macrophages through exosomes, which may contribute to the formation of immunosuppressive microenvironments. Taken together, our study reveals the miR-125a-STAT3 pathway through which exosomal NEAT1 from treatment-resistant GSCs contributes to M2-like macrophage polarization, indicating the potential of exosomal NEAT1 for treating glioma.

## 1. Introduction

Gliomas emerge as highly aggressive neurological malignancies for the central nervous system (CNS) [1], and the median survival of glioblastoma does not exceed 2 years despite maximal surgical resection followed by alkylating drug temozolomide (TMZ) combined with concurrent aggressive radiotherapy and subsequent chemotherapy [2]. The main reason is related to the endurable responses to radio and chemotherapy of the patient’s body, with resistance to radiotherapy and chemotherapy elicited after a period of radio and chemotherapy. Glioma stem cells (GSCs) and their immunosuppressive microenvironment prominently affect resistance to radiotherapy and chemotherapy that directly induces tumor recurrence and shortened survival time [3].

GSCs are characterized by unlimited self-renewal, proliferation, differentiation into different mature tumor cells, and high tumorigenic potential [4]. Resistance to chemotherapy and radiation is considered a crucial trait of cancer stem cells (CSCs) [5,6,7]. Additionally, the immunosuppressive microenvironment also plays an essential role in glioma chemoradiotherapy resistance, and the immunosuppressive microenvironment acts as the main challenge for the inefficacy of immunotherapy. Increasingly, studies have demonstrated tumor-associated macrophages (TAMs) as the primary immune cells that infiltrate the glioma microenvironment to promote glioma progression [8]. With the significant correlation to glioma progression, grade, and patient prognosis of TAMs, glioma-associated macrophages (GAMs) play a particularly important role in glioblastoma [9,10], in addition, the colocation of GSCs with tumor hypoxic and perivascular environments suggests a prominent role of their functional interactions in tumorigenesis [11]. Notably, it has been reported that glioma progression and tumor growth could be prevented by blocking the TAM polarization into the M2 subtype or reducing the number of TAMs by eliminating macrophages [12,13]. It is certain that the functional correlation of TAMs and GSCs is convincing considering the crosstalk between them, while the molecular link has remained to be defined. The emerging evidence has validated the participation of exosomes in this progress by transferring their contents to recipient cells [14]. In recent years, exosomes, considered a new means of cell communication, have raised extensive attention due to their capability to carry various contributing information such as proteins, lipids, micro-RNAs (miRNAs), and long noncoding RNAs (lncRNAs) [15], which have been shown to exert diverse functions in tumor development and genesis. For instance, the enriched PRPS2 in the exosomes of non-small-cell lung carcinoma (NSCLC) cells mediates the polarization of M2 macrophages to promote the cisplatin resistance of non-small-cell lung cancer (NSCLC) cells [16]. As a carrier of a variety of bioactive molecules, exosomes can easily cross biological barriers such as the blood–brain barrier (BBB), which gives great hope and prospects for clinical treatment [17,18]. Up to now, the role of GSC-derived exosomes in TAMs remains to be elucidated.

Long noncoding RNAs (lncRNAs) transcribed throughout the genome have been a recently discovered class of noncoding genes that have been suggested to exert functions in modifying multiple elements of tumor biology in response to external factors or DNA damage. Radiotherapy, chemotherapy, and DNA damage stimulation could elevate the expression of lncRNA-NEAT1 in glioma cells [19,20,21]. NEAT1 is reported to be overexpressed in GSCs, and the expression of stemness-related transcription factors could be reduced by silencing the expression of NEAT1. In addition, inhibiting NEAT1 in GSCs could reduce tumorsphere formation and weaken their proliferation, invasion, and migration [22,23].

This study is conducted to explore the effect of GSC-derived exosomes on the recruitment, polarization, and function of TAMs, as well as to investigate the role of NEAT1 in the function of GSC-derived exosomes. This study provides evidence that exosomes carrying NEAT1 may serve as a potential therapeutic target for glioma therapy.

## 2. Materials and Methods

### 2.1. Cell Lines

Human GBM cell lines (U251, A172) were obtained from the ATCC (American Type Culture Collection), while the mice GBM cell line GL261 was kindly provided by Dr. Zhao (State Key Laboratory of Cancer Biology, Department of Medical Genetics and Developmental Biology, Air Force Military Medical University). The U251, A172, and GL261 cells were cultured in Dulbecco’s modified eagle’s medium (DMEM, Gibco, Grand Island, NY, USA) supplemented with 10% fetal bovine serum (FBS, Hyclone, Logan, UT, USA) at 37 °C in a humidified environment with 5% CO_2_, which then tested negative for mycoplasma contamination. Macrophages (BMDMs) derived from mouse bone marrow were generated as previously described [24]. In brief, 4-week-old C57BL/6 mice were killed through cervical dislocation and dissected to obtain both femurs free of adherent tissue. The marrow tissue was eluted by irrigation with PBS after discarding the ends of the bones. Cells were suspended by vigorous pipetting, washed once by PBS, collected through centrifugation, and then cultured in bone marrow growth medium (50% DMEM, 20% fetal calf serum, 30% L929-conditioned medium, and 1x penicillin–streptomycin–glutamine) for 8–10 days.

### 2.2. Induction of Glioma Stem Cells

The glioma stem cells were cultivated in neural stem cell (NSC) medium, which contained DMEM/F12 with high glucose (Gibco, Grand Island, NY, USA), 10 ng/mL bFGF (Invitrogen, Waltham, MA, USA), 10 ng/mL EGF (Invitrogen, Waltham, MA, USA), B27 (Gibco, Grand Island, NY, USA), maintained at 37 °C in 5% CO_2_ and 95% air. The medium was replaced every 3–4 days. And the glioma stem cell spheres were observed after 8–10 days of cultivation.

### 2.3. siRNA Construction and Infection

Cells were transfected with siRNAs using Lipofectamine 2000 (Invitrogen, Waltham, MA, USA). The sequences of siRNAs against specific targets are provided in Table S1.

### 2.4. RNA Extraction and Quantitative Real-Time Polymerase Chain Reaction (qRT-PCR)

The total RNA of GBM tissues and cancer cells was extracted by TRIzol Reagent (Invitrogen, Waltham, MA, USA), with the concentration and purity measured by Eppendorf Biophotometer plus. The PrimeScript™ RT Reagent Kit (Takara, Kusatsu, Japan) was employed to reverse transcribe lncRNA and mRNA. The relative gene expression levels were determined by the SYBR Green PCR Kit (Takara, Kusatsu, Japan). The relative quantity of gene expression was calculated taking the standard 2^−∆∆Ct^ with GAPDH or U6 as an internal control. The sequences of the primers are listed in Table S2.

### 2.5. Immunofluorescence Experiment

In order to dewax paraffin sections, the paraffins were placed in sections in a 70 °C baking machine and heated for 30 min and then immersed in xylene A (15 min), xylene B (15 min), xylene C (15 min), anhydrous ethanol A (5 min), anhydrous ethanol B (5 min), 95% ethanol A (5 min), 95% ethanol B (5 min), 90% ethanol (3 min), 80% ethanol (3 min) in sequence. After rinsing with tap water for 3 min, paraffin sections were repaired at high temperature by EDTA-Tris antigen repair solution in a PH = 9.0. The staining of primary antibodies (Table S3) was maintained overnight, and the secondary antibodies were maintained for 3 h. Finally, images were observed and collected through an inverted fluorescence microscope.

### 2.6. Protein Preparation and Western Blotting

The total proteins prepared from GBM cells were obtained from prechilled RIPA buffer with proteinase and phosphatase inhibitor cocktails (Selleck.cn, Shanghai, China). The PVDF membranes were incubated with primary antibodies overnight (Table S3) at 4 °C and then with an HRP-labeled secondary antibody (Zsbio Store-bio, Beijing, China) at room temperature for 1 h. The protein bands were visualized depending on a Chemiluminescence Reagent (ECL) Kit (Boster, Wuhan, China).

### 2.7. In Vivo Xenograft Model

The 6-week-old female C57BL/6 mice were purchased from the Air Force Medical University Animal Center (Xian, China). GL261 cells or GL261-GSCs cells at the logarithmic growth stage were digested with 0.1% trypsin (Invitrogen, Waltham, MA, USA) and then resuspended with PBS, and cell counts were performed. The cell concentration was adjusted to 6.66 × 10^7^/mL. After anesthesia, the mice were fixed in a prone position, and the cells were absorbed with a microsyringe and fixed vertically above the skull of the mice. Before inoculation, the skin on the top of the mice was disinfected with 75% alcohol and cut open to expose the skull, and the bare fontanel was found. The insertion point was located 2.5 mm to the right of the fontanel and 0.5 mm to the anterior side with a stereotaxic device. The outer part of the skull was worn away but not penetrated by a cranial drill at the insertion point. Gently prick the remaining thin skull layer with a microsyringe and continue to insert the needle 2.5 mm from the skull. Adjust the injection rate of the microinjection instrument to 2 μL/min, and the total injection volume is 15 μL (1 × 10^6^ cells). Skull openings were sealed with bone wax, followed by skin sutures and disinfection. Mice were humanely sacrificed by sodium pentobarbital 21 days after tumor inoculation and the tumor tissues for the following experiment were removed.

### 2.8. Exosome Isolation

GL261 cells and GL261 stem cells were cultured in a suitable medium for 48 h. The medium was collected after cell counting for exosome isolation. A serial centrifugation procedure (300× *g* for 10 min, 2000× *g* for 10 min, and 10,000× *g* for 30 min) was carried out to collect the supernatant after each centrifugation using a 0.22 µm pore, followed by a spin at 100,000× *g* for 70 min. The collected pellet was washed in PBS three times before another ultracentrifugation at 100,000× *g* for 60 min. The exosomes were collected for further experiments.

### 2.9. Flow Cytometry

To detect the expression of macrophage producers, a single-cell suspension was obtained from well-grown attached cells by trypsinization (without EDTA) under sterile conditions and stained with IgG1 Isotype Control Summary (R&D Systems, Minneapolis, MN, USA) for about 15 min at 4 °C in the dark. Afterward, cells were washed in cold PBS, centrifuged at 1000 rpm for 5 min at 4 °C, and subsequently stained with the antibodies for about 30 min at 4 °C in the dark. Fluorescence-activated cell sorting (FACS) CantoII Flow Cytometer (BD Biosciences, Franklin Lakes, NJ, USA) was performed to examine the results.

### 2.10. Transmission Electron Microscope

Suspension drops of 20 uL were absorbed by pipette from the prepared suspension samples of exosomes to the copper net for natural adsorption for 5–10 min, with the excess drops wiped off by filter paper and then dried slightly. A 2% phosphotungstic acid solution of 20 uL was dropped on the copper net and stood for 3–5 min. The excess droplets were absorbed by filter paper and dried under an incandescent lamp. A transmission electron microscope was used to observe and take photos.

### 2.11. Nitric Oxide (NO) Detection

The release of nitric oxide (NO) (Griess reaction) was detected in culture media supernatants using the NO assay kit (Solarbio, Beijing, China) according to the manufacturer’s protocols. Briefly, 264.7 cells were seeded in 96-well plates at a density of 2 × 10^4^ cells/well. After the corresponding treatment, 100 mL of supernatants of BMDMs were collected and mixed with 20 μL Griess reagent I and II in a new 96-well plate. After the color reagent was added, the NO concentrations were determined at 540 nm using BIO-RAD Microplate Reader Model 680 (BIO-RAD, Shanghai, China).

### 2.12. Luciferase Reporter Assay

BMDMs were seeded into 24-well plates and cultured overnight, and miR-125a wild-type cells with potential STAT3 binding sites or mutants of each binding site were generated and fused to the luciferase reporter pGL3-basic plasmid, alone or following pretreatment with miR-125a mimics or inhibitor. According to the manufacturer’s protocol, the relative luciferase activity in each well was analyzed after 24 h using a dual-luciferase reporter assay system (Promega, Madison, WI, USA).

### 2.13. Statistical Analysis

All statistical analyses were performed on SPSS 19.0 (IBM Corporation, Endicott, NY, USA) and GraphPad Prism9.0 (GraphPad Software Inc., Boston, MA, USA). Student’s *t*-test or standard one-way ANOVA was performed to test the intergroup differences. At least three separate in vitro studies were conducted, with the data described as means ± standard deviations (SD). Statistical significance was indicated.

## 3. Results

### 3.1. GL261 Cells and GL261 GSCs Were Inoculated Intracranially in C57BL/6 Mice

**Figure 1:**
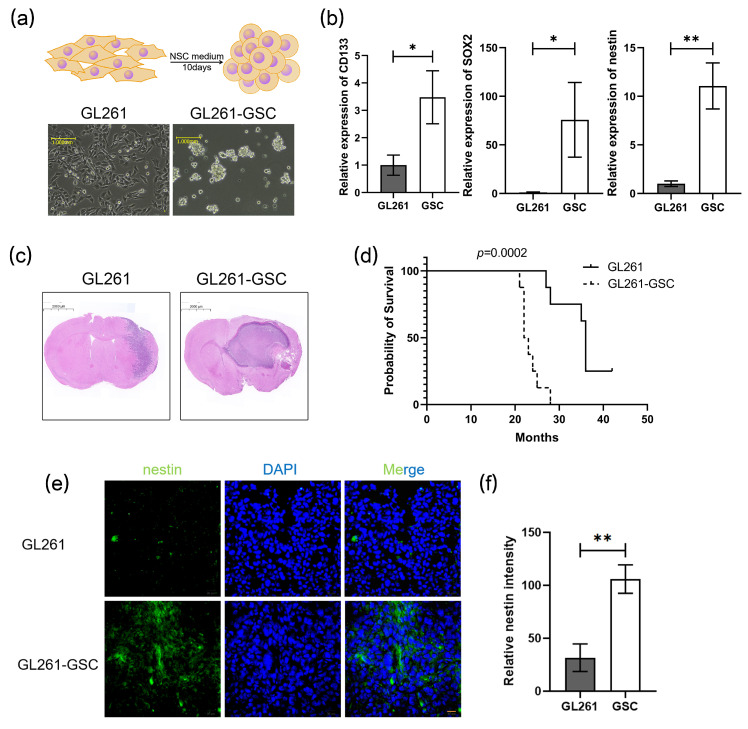
GL261 cells and GL261 GSCs were inoculated intracranially in C57BL/6 mice: (**a**) upper: GL261 cells were induced into GSCs; lower: representative images of GL261 cells and GSCs tumorspheres are shown; (**b**) QRT-PCR analysis of the expression of several stem cell markers in GL261 and GL261-GSCs. GAPDH is used as a reference gene (*n* = 3); (**c**) HE staining image of mice tumor section; (**d**) Kaplan–Meier survival curve of mice with GL261 brain tumor (*n* = 10) or GL261-GSC (*n* = 10) brain tumor in our study is shown; (**e**) representative immunofluorescence images of glioma sections that were obtained from different groups of mice and stained for the stem cell marker nestin (green) and DAPI (blue) are shown; (**f**) graphical analysis of nestin is shown (*n* = 3). Unless otherwise noted, data are presented as the mean ± S.D. *p*-value is determined by Student’s *t*-test (* *p* < 0.05, ** *p* < 0.01).

GL261 cells were induced into GL261 stem cells after being cultured in an NSC medium for 10 days (referred to as GL261-GSC). The stemness properties of GL261-GSCs were evaluated according to their capability to form neurospheres (Figure 1a) and the expression of stem cell markers, CD133, SOX2, and nestin (Figure 1b). C57BL/6 mice were orthotopically administered by GL261 or GL261-GSCs, named GL261 mice or GSCs mice. After 21 days of cell inoculation, the mice’s brains were removed and sectioned at the insertion site to show the HE-dyed pictures. The tumors of the GSC group mice exhibited enlarged volumes (Figure 1c) and a significantly reduced life span (Figure 1d). The immunofluorescence assay indicated more aggregated nestin in the GSCs tumor nests (Figure 1e,f), which illustrated the maintained stemness of GL261-GSCs in vivo.

### 3.2. The Number of M2-like TAMs Increased in the GSCs Niches

**Figure 2:**
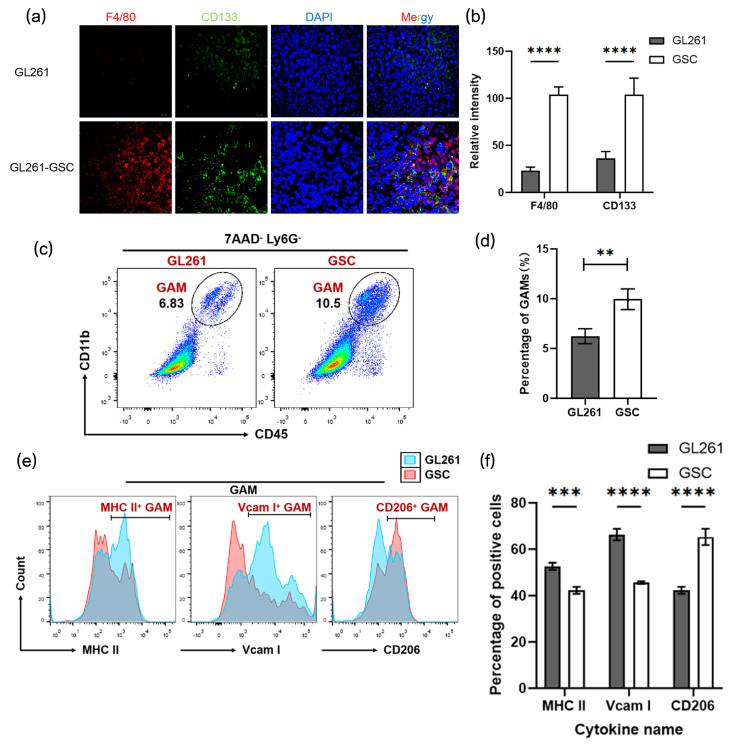
The number of M2-like TAMs increased in the GSC niches: (**a**) representative immunofluorescence images of glioma sections that were obtained from different groups of mice and stained for the macrophage markers CD206 (red), stem cell marker nestin (green), and DAPI (blue) are shown; (**b**) graphical analysis of CD206 and nestin are shown (*n* = 3); (**c**) flow cytometric analysis of CD11b+ CD45+ 7AAD− Ly6G− macrophages in tumors from different groups of mice; (**d**) the statistical result of the flow cytometric analysis is shown (*n* = 3); (**e**) flow cytometric analysis of MCH II +, Vcam I+, and CD206+ macrophages in tumors from different groups of mice; (**f**) the statistical result of the flow cytometric analysis is shown (*n* = 3). Unless otherwise noted, data are presented as the mean ± S.D. *p*-value is determined by Student’s *t*-test (** *p* < 0.01, *** *p* < 0.001, **** *p* < 0.0001).

The macrophage marker F4/80 and the stem cell marker CD133 were employed to identify the macrophage composition of tumors. The increased F4/80+ macrophage infiltration found in GSC mice tumor tissue was almost surrounded by CD133+ cells (Figure 2a,b). Tumor tissues isolated from GL261/GL261-GSCs mice were digested into a single-cell suspension and subjected to FACs, which revealed the significantly higher percentage of GAMs (CD11b+ CD45+ 7AAD− Ly6G−) cells in GSC tumor tissues (Figure 2c,d and Figure S1). In addition, it was the population of M2-type macrophages (CD206+), not M1-type macrophages (MHC II+, Vcam I+), that occupied most of the GAMs (Figure 2e).

### 3.3. GL261-GSC-Derived Exosome Induces Macrophage M2 Polarization

**Figure 3:**
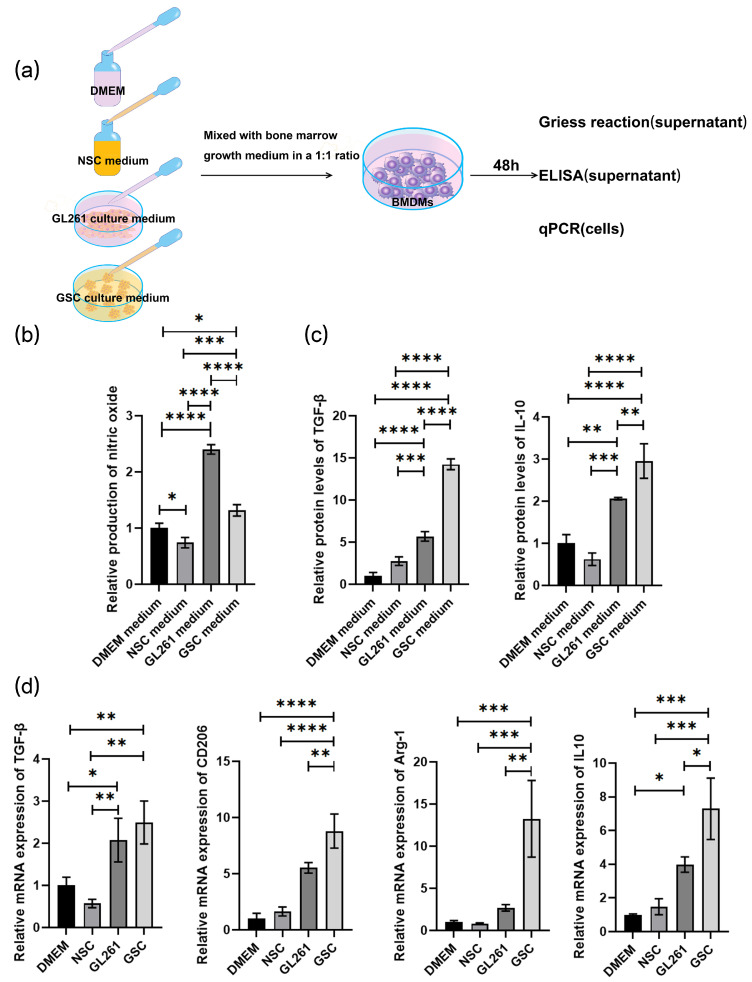
GL261-GSCs induce macrophage M2 polarization: (**a**) BMDMs were cocultured with blank DMEM medium, blank NSC medium, culture medium of GL261 cells, and culture medium of GL261-GSCs; (**b**) relative quantitative results of the Griess experiment in the culture medium of four groups of BMDMs (BMDMs cultured with blank DMEM medium, blank NSC medium, culture medium of GL261 cells, and culture medium of GL261-GSC cells) (*n* = 3); (**c**) relative quantitative results of ELISA experiment of TGF-β and IL10 protein levels in the culture medium of four groups of BMDMs (BMDMs cultured with blank DMEM medium, blank NSC medium, culture medium of GL261 cells, and culture medium of GL261-GSC cells) (*n* = 3); (**d**) relative quantitative results of qRT-PCR analysis of the expression TGF-β, CD206, Arg-1, and IL-10 in four groups of BMDMs (BMDMs cultured with blank DMEM medium, blank NSC medium, the culture medium of GL261 cells, and the medium of GL261-GSC cells) (*n* = 3). Data are presented as mean ± S.D. *p*-value is determined by ordinary one-way ANOVA (* *p* < 0.05, ** *p* < 0.01, *** *p* < 0.001, **** *p* < 0.0001).

**Figure 4:**
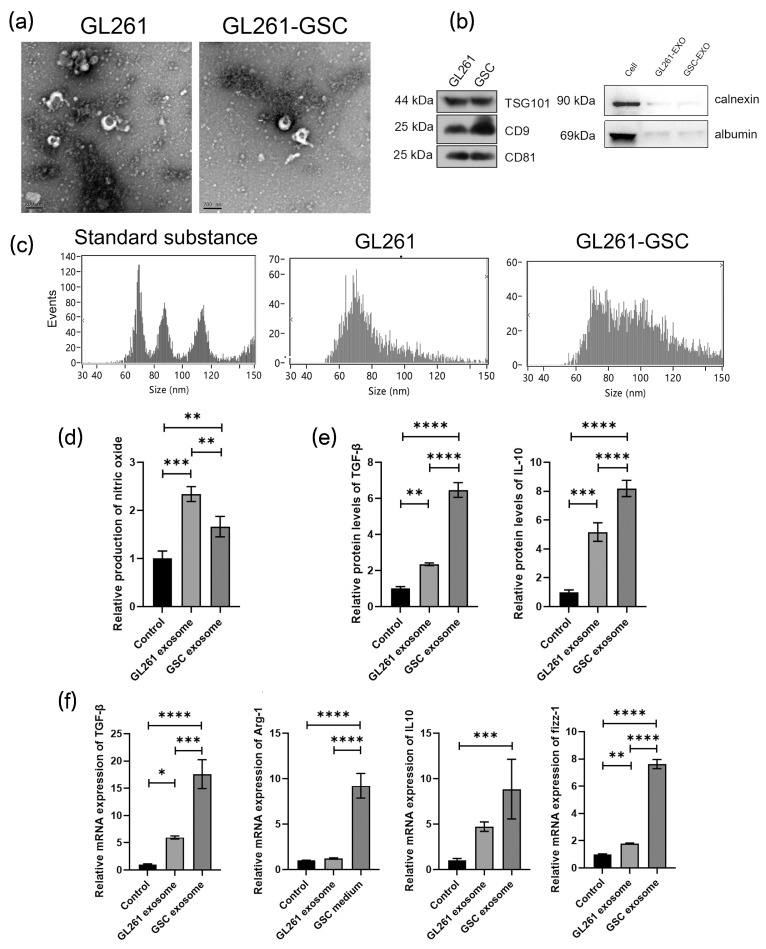
GL261-GSCs-derived exosomes induce macrophage M2 polarization: (**a**) representative image from an electron microscope of exosomes collected from the culture medium of GL261 and GL261-GSC cells; (**b**) proteins are extracted from the exosomes of GL261 cells and the expression of CD9, CD81, TSG101, calnexin, and albumin is determined by Western blot analysis; (**c**) particle size map of exosomes from GL261 and GL261-GSC cells, and standard substance is used as a comparison; (**d**) relative quantitative results of the Griess experiment in the culture medium of BMDMs cultured with the exosomes from GL261 or GL261-GSC cells (*n* = 3); (**e**) relative quantitative results of ELISA experiment of TGF-β and IL10 protein levels in the culture medium of BMDMs cultured with the exosomes from GL261 or GL261-GSC cells; (**f**) relative quantitative results of qRT-PCR analysis of the expression TGF-β, CD206, Arg-1, and IL-10 in BMDMs cultured with the exosomes from GL261 or GL261-GSC cells. GAPDH is used as a reference gene (*n* = 3). Data are presented as the mean ± S.D. *p*-value is determined by ordinary one-way ANOVA (* *p* < 0.05, ** *p* < 0.01, *** *p* < 0.001, **** *p* < 0.0001).

BMDM macrophage cells were initially cocultured in the culture medium of GL261 cells and GL261-GSCs (Figure 3a). The Griess reaction indicated the significantly weakened NO production capacity of BMDMs cocultured with GL261-GSCs compared with the GL261 group (Figure 3b). The ELISA test demonstrated the promoted TGF-β and IL-10 protein levels in the supernatants of the GL261-GSCs group (Figure 3c). In addition, the GSCs medium promoted the expression of the mRNA levels of M2-type macrophage producers, TGF-β, Arg-1, CD206, and IL-10 in BMDMs (Figure 3d). To examine the role of exosomes in macrophage functional remodeling, exosomes were isolated from the culture medium of GL261 cells and GL261-GSCs and identified by electron microscope, as well as different markers of exosomes CD9, CD81, and TSG101, negative markers calnexin and albumin, and the particle size distribution (Figure 4a–c). Next, these two groups of exosomes were cocultured with BMDMs, respectively. The Griess reaction, ELISA, and qRT-PCR consistently pointed to a definite trend of functional remodeling and a trend towards M2 polarization of the BMDMS cocultured with GL261-GSCs exosomes (Figure 4d–f). Taken together, it is speculated that the secretion of exosomes by GSCs contributes to remodeling the glioma microenvironment and promotes the M2 polarization of macrophages.

### 3.4. Reduced Secretion of Exosomes Alters Macrophages Phenotypes

**Figure 5:**
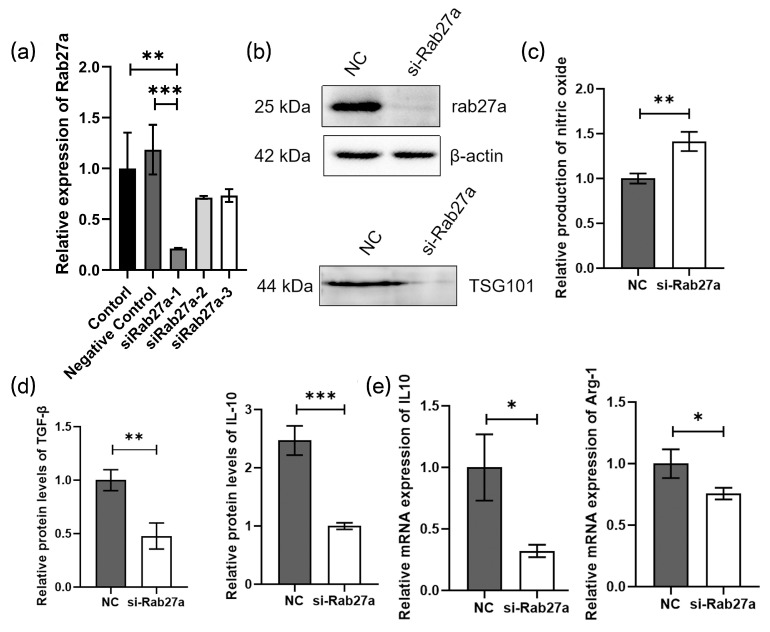
Reduced secretion of exosomes alters macrophage phenotypes: (**a**) Rab27a siRNAs are transfected into the GL261-GSC cells and then the expression of rab27a is assessed by qRT-PCR analysis. GAPDH is used as a reference gene (*n* = 3); (**b**) the expression of Rab27a and exosomes marker TSG101 was detected by Western blot; (**c**) relative quantitative results of the Griess experiment of in the culture medium of BMDMs cultured with the culture medium of si-NC or siRab27a-GL261-GSC cells (*n* = 3); (**d**) relative quantitative results of ELISA experiment of TGF-β and IL10 protein levels in the culture medium of BMDMs cultured with the culture medium of si-NC or siRab27a-GL261-GSC cells (*n* = 3); (**e**) relative quantitative results of qRT-PCR analysis of the expression IL-10 and Arg-1 in BMDMs cultured with the culture medium of si-NC or siRab27a-GL261-GSC cells. GAPDH is used as a reference gene (*n* = 3). Data are presented as the mean ± S.D. *p*-value is determined by Student’s *t*-test or ordinary one-way ANOVA (* *p* < 0.05, ** *p* < 0.01, *** *p* < 0.001).

The expression of Rab27a has been demonstrated to manipulate exosome release [25,26]. To examine the role of exosomes in the recruitment and induction of TAMs, the Rab27a were knocked down by Rab27a siRNA transfection in GL261 GSCs and verified by qPCR and Western blot (Figure 5a,b), and a decrease in exosomal markers TSG101 after Rab27a silencing was observed (Figure 5b). BMDMs were cocultured with the culture medium of two group cells (GSC-NC, GSC-siRab27a), and those with GSC-siRab27a medium were found to produce higher levels of NO (Figure 5c). The TGF-β and IL10 ELISA and qPCR detection of IL-10, Arg-1 also demonstrated a decline in Rab27a-deficient GSCs in their capability to induce macrophage M2 polarization (Figure 5d,e).

### 3.5. NEAT1 Delivered by Exosomes Promotes the M2 Polarization of Macrophages by Target miR-125a/STAT3 Axis

**Figure 6:**
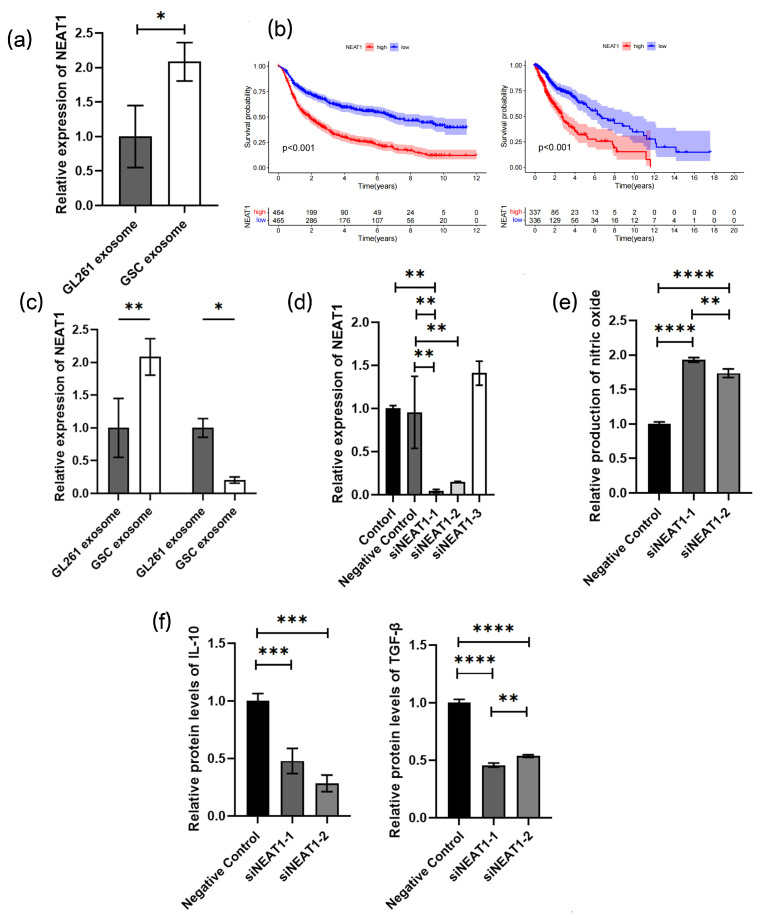
NEAT1 delivered by exosomes promotes the M2 polarization of macrophages: (**a**) relative quantitative results of qRT-PCR analysis of the expression NEAT1 in exosomes from GL261 or GL261-GSC cells. GAPDH is used as a reference gene (*n* = 3); (**b**) Kaplan–Meier survival curves of NEAT1 high-expression and low-expression glioma patients were obtained from CGGA (right, http://www.cgga.org.cn/, accessed on 3 February 2024) or TCGA (Left, https://www.cancer.gov/about-nci/organization/ccg/research/structural-genomics/tcga, accessed on 3 February 2024) databases, and the included patients and their gene expression and survival time are listed in the supplemental materials; (**c**) relative quantitative results of qRT-PCR analysis of the expression NEAT1 and miR-125a in BMDMs cultured with the exosomes from GL261 or GL261-GSC cells. GAPDH or U6 is used as a reference gene (*n* = 3); (**d**) NEAT1 siRNAs are transfected into the BMDMs and then the expression of NEAT1 is assessed by qRT-PCR analysis. GAPDH is used as a reference gene (*n* = 3); (**e**) relative quantitative results of the Griess experiment in the culture medium of si-NC or siNEAT1 BMDMs (*n* = 3); (**f**) relative quantitative results of ELISA experiment of TGF-β and IL10 protein levels in the culture medium of si-NC or siNEAT1 BMDMs (*n* = 3). Data are presented as the mean ± S.D. *p*-value is determined by Student’s *t*-test or ordinary one-way ANOVA (* *p* < 0.05, ** *p* < 0.01, *** *p* < 0.001, **** *p* < 0.0001).

**Figure 7:**
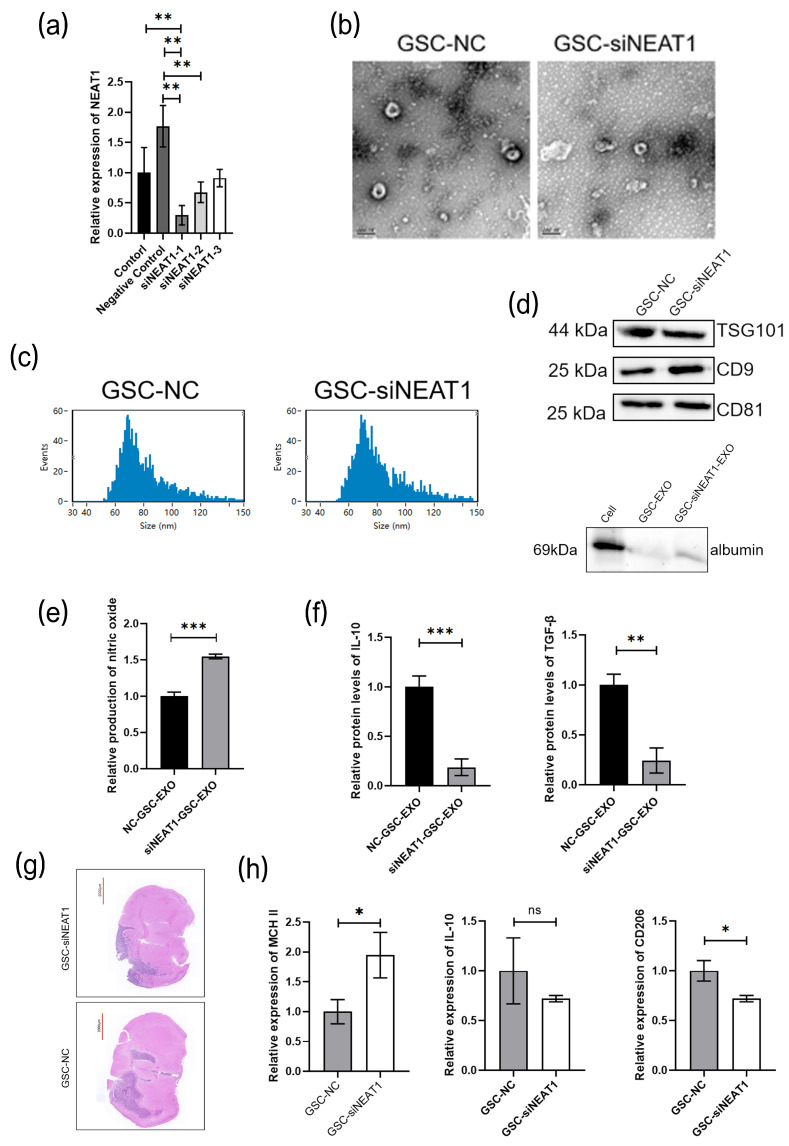
(**a**) NEAT1 siRNAs are transfected into the GSCs and then the expression of NEAT1 is assessed by qRT-PCR analysis. GAPDH is used as a reference gene (*n* = 3); (**b**) representative image of electron microscope of exosomes collected from the culture medium of GSC-NC and GSC-siNEAT1 cells; (**c**) particle size map of exosomes from GSC-NC and GSC-siNEAT1 cells. (**d**) Proteins are extracted from the exosomes of GSC-NC and GSC-siNEAT1 and the expression of CD9, CD81, TSG101, and albumin are determined by Western blot analysis; (**e**) relative quantitative results of the Griess experiment in the culture medium of GSC-NC-EXO and GSC-siNEAT1-EXO (*n* = 3); (**f**) relative quantitative results of ELISA experiment of TGF-β and IL10 protein levels in the culture medium of GSC-NC and GSC-siNEAT1 cells (*n* = 3); (**g**) HE staining image of mice tumor section; (**h**) relative quantitative results of qRT-PCR analysis of the expression MCH II, IL10, and CD206 in macrophages in tumors from different groups of mice;. GAPDH is used as a reference gene (*n* = 3). Data are presented as the mean ± S.D. *p*-value is determined by Student’s *t*-test or ordinary one-way ANOVA (* *p* < 0.05, ** *p* < 0.01, *** *p* < 0.001, ns = not significant).

**Figure 8:**
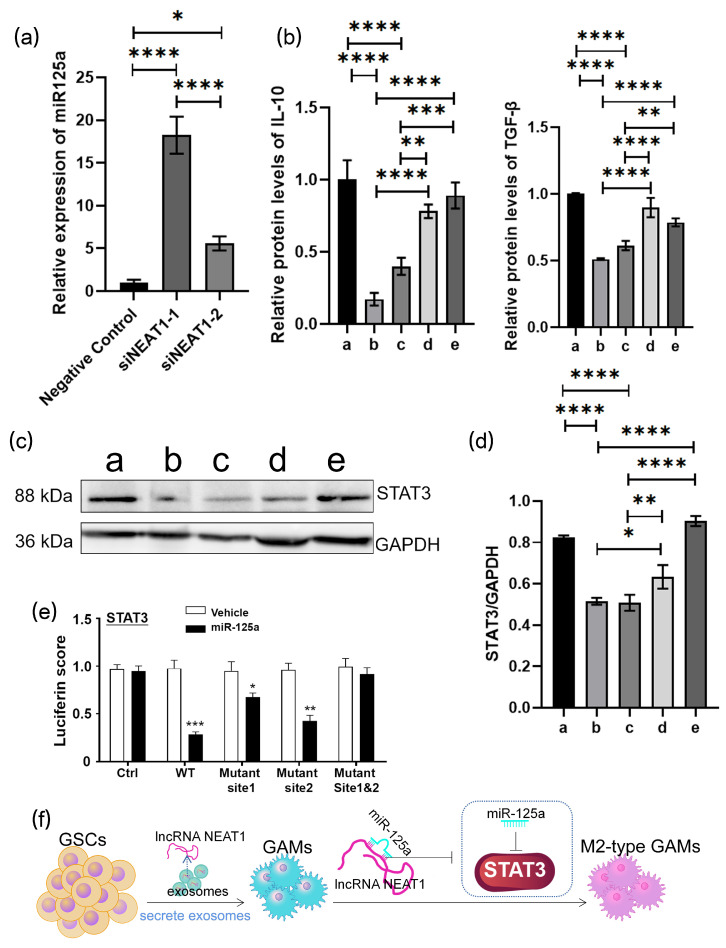
NEAT1 delivered by exosomes promotes the M2 polarization of macrophages: (**a**) relative quantitative results of qRT-PCR analysis of the expression miR-125a in si-NC or siNEAT1 BMDMs. U6 is used as a reference gene (*n* = 3); (**b**) relative quantitative results of ELISA experiment of TGF-β and IL10 protein levels in the culture medium of different groups of BMDMs (a: negative control, b: siNEAT1-1, c: siNEAT1-2, d: siNEAT1-1+miR125a inhibitor, e: siNEAT1-2+miR125a inhibitor) (*n* = 3); (**c**) protein levels of STAT3 of different groups of BMDMs are determined by Western blot (a: negative control, b: siNEAT1-1, c: siNEAT1-2, d: siNEAT1-1+miR125a inhibitor, e: siNEAT1-2+miR125a inhibitor); (**d**) quantitative results of Western blot analysis are shown (*n* = 3); (**e**) MiR-125a mimics and reporter plasmids containing the wild type or mutant form of STAT3 3′UTR are co-transfected into the BMDMs, and then the luciferase activity is assessed after 48 h transfection. Expression of STAT3 is shown. (**f**) The mechanistic scheme of GSC-derived exosomes remodeled GAMs by delivering lncRNA NEAT1 to GAMs and regulating the NEAT1/miR-125a/STAT3 pathway. Data are presented as the mean ± S.D. *p*-value is determined by ordinary one-way ANOVA (* *p* < 0.05, ** *p* < 0.01, *** *p* < 0.001, **** *p* < 0.0001).

A dysregulation of NEAT1 has been revealed in various human cancer specimens, which may serve as a potential biological factor for tumor diagnosis and prognosis [27,28]. Human GBM cell lines U251 and A172 were also induced into U251-GSC and A172-GSC, and we detected NEAT1 expression in GL261/GL261-GSC, U251/U251-GSC, and A172/A172-GSC exosomes and found an upregulated expression in all GSC cell lines (Figure 6a, Figure S3). According to the TCGA and CGGA databases, a negative correlation of the NEAT1 expression with the survival outcome of glioma patients was revealed (Figure 6b). Studies have evidenced that silencing NEAT1 could upregulate the miR-125a and elicit the M2 polarization of macrophages [29]. We found a significantly increased expression of NEAT1 and a significantly decreased expression of miR-125a in BMDMs after coculturing with exosomes of GL261-GSCs. (Figure 6c). The expression of NEAT1 was knocked down in BMDMs (Figure 6d), and the silencing of NEAT1 could promote the M2 polarization of macrophages according to further Griess assay and ELISA assay (Figure 6e,f). To further examine the role of NEAT1 in macrophage functional remodeling, the expression of NEAT1 was knocked down in GSC cells, and exosomes were isolated from the culture medium of GSC-NC and GSC-siNEAT1 cells and identified by electron microscope, particle size distribution, and different markers of exosomes CD9, CD81, and TSG101 and negative markers of albumin. BMDMs were cocultured with the culture medium of two group exosomes (GSC-NC and GSC-siNEAT1), and those with GSC-NEAT1 exosomes were found to produce higher levels of NO (Figure 7a–d). The TGF-β and IL10 ELISA demonstrated a decline in GSC-si-NEAT1 in the capability to induce macrophage M2 polarization (Figure 7e,f). C57BL/6 mice were orthotopically administered by GSC-NC and GSC-siNEAT1, and the tumors of the GSC group mice exhibited enlarged volumes (Figure 7g). Macrophages were isolated by flow cytometry, and the PCR assay indicated more aggregated M1-type macrophages occupied most of the GAMs (Figure 7h). Subsequently, miR-125a expression was found to be significantly promoted after NEAT knockdown (Figure 8a). ELISA assay indicated the rescued decreased protein expression of IL-10 and TGF-βby miR-125a inhibitor (Figure 8b). The Western blot assay further indicated a decrease in STAT3 expression due to the downregulation of NEAT1, which could be partially upregulated by the miR-125a inhibitor (Figure 8c,d). A luciferase reporter assay was performed to determine whether STAT3 is indeed a target of miR-125a through the 3′ UTR region. The results reveal that the upregulation of miR-125a resulted in the deactivation of the 3′ UTR of STAT3 (Figure 8e). Conclusively, NEAT1 delivered by exosomes may influence the phenotype of TAMs, and NEAT1 could target the miR-125a/STAT3 axis in macrophages to regulate macrophage functioning (Figure 8f).

## 4. Discussion

CSCs, that is, tumor-initiating cells, are considered the drivers of tumor growth and recurrence that generally exist in heterogeneous, invasive, and treatment-resistant tumors [7]. Despite the thoroughly described mechanisms of CSCs for tumor growth, recurrence, and drug resistance, the origin of GSCs is not fully illustrated. According to some studies, CSCs can emerge in response to chemotherapy and TME disorders, which are suggested to be derived from non-CSC tumor cells [30]. It has also been reported that GSCs could be sourced from malignantly transformed NSCs or neural progenitor cells and transdifferentiated mature glial cells [31,32]. More precisely, the development and function of GSCs, at least, are partially dependent on the microenvironment. Maintaining the status of GSCs in the glioma microenvironment requires signal exchange and interaction at multiple levels, not only the interaction between tumor cells but also between GSCs and nontumor cells in the microenvironment [33,34,35,36,37]. Brain tumor microenvironment (TME) refers to a mixture of tumor cells and endogenous host stroma such as microglia/macrophages, endothelial cells, astrocytes, and glioma stem cells (GSCs) [38], where glioma-associated macrophages (GAMs) serve as a major component that depends on the activation of microglia residing in intracranial tumors and the immunomodulatory activity of macrophages [9]. The phenotypes of macrophage could be divided into two categories, the classically activated macrophages (Ml type) and alternatively activated macrophages (M2 type) [39]. The studies that are available demonstrate that the Ml type triggers TH1 proinflammatory responses that can assault cancer cells and restore normalcy to atypical neovascular networks, enhancing treatment sensitivity [40]. The M2 type associated with an anti-inflammatory TH2 immune response plays a role in tumor progressions by promoting angiogenesis, microenvironmental remodeling, ectopic tumor growth, and immunosuppression [41]. It is increasingly uncovered that TAMs generally exhibiting the M2 phenotype lack apparent phagocytic activity [42].

GAMs serve as the primary immune cells for invading the glioma microenvironment and promoting glioma progression, accounting for 30–40% of the total number of cells in the tumor microenvironment [43]. The number of GAMs shows a correlation with the proliferation of glioma cells, tumor grade, and the prognosis of glioma patients [44]. Notably, GAMs mainly surround the microvessels and hypoxic regions, accompanied by a spatial adjacency to the NESTIN-positive and CD133-positive GSCs. Studies have confirmed a colocation of GSCs and GAMs in the specific stem cell niche, which promotes the malignant progression of glioma by influencing each other [45]. Zhou et al. demonstrated an involvement of GSCs in the modification of the immunosuppressive microenvironment by secreting periostin (POSTN) to recruit M2-type TAMs and promote GBM progression and tumor recurrence [45]. In addition to metabolites and chemokines, exosomes also participate in the dynamic communication between GSC and GAMs to contribute to the malignant progression of gliomas.

Exosomes carry a variety of biological materials that can easily cross biological barriers and active molecules, such as the BBB, which has raised great hope and prospects for clinical treatment [17]. The in vitro study of gliomas showed a modification of U87MG-derived exosomes on blood-derived monocytes into M2-like macrophages [46]. We displayed the increased distribution of GSCs and M2-like GAMs (GAMs) in the GSCs niche and the number of M2-like GAMs in the GSCs niches in C57BL/6 mice inoculated with GL261-GSCs intracranially. Further study demonstrated that the secretion of exosomes by GSCs contributes to remodeling the glioma microenvironment by modulating the polarization of macrophages.

The hypoxic glioma-derived EV-mediated delivery of Mir-1246 polarizes the macrophages toward M2 by inhibiting NF-κB and activating the STAT3 pathway [47]. However, miRNAs are easily degraded by extensive pairing to some unusual target RNAs due to their instability [48]. LncRNAs with a length over 200 bp are relatively more stable compared with other RNAs, which makes lncRNAs carried by exosomes considered effective therapeutic agents. It is becoming increasingly clear that the usage of chemotherapy or other targeted medications during treatment can promote the formation of resistant cells that resemble CSCs [49]. The development of GSCs is regarded as one of the primary causes of temozolomide (TMZ) resistance in glioma patients, and TMZ treatment could enhance GSC production by glioma cells [20]. It has also been reported that DNA damage, chemotherapy, and radiotherapy could elicit the upregulated expression of lncRNA-NEAT1 in glioma cells [50]. NEAT1 can also promote the malignant biological influences of GSCs. Our study confirmed the highly expressed lncRNA NEAT1 in exosomes from GL261-GSCs compared with exosomes from GL261. There is sufficient evidence that NEAT1 directly targets miR-125a as a competing endogenous RNA (ceRNA) [29,51]. Our study demonstrated that lncRNA NEAT1 targets miR-125a to promote M2 macrophage polarization by directly upregulating STAT3 in glioma. Since the molecular mechanisms and functions of endogenous miRNAs released in response to various modalities in GSCs are widely understood and researched, for example, glioma-released miR-1983 within exosomes could initiate an antiglioma NK-mediated circuit [52], our findings may potentially provide novel targets for designing therapeutic strategies.

## 5. Conclusions

In summary, our study demonstrated the increased aggregation of macrophages, especially M2-type macrophages, in glioma carcinoma nests. The GSC-derived exosomes are closely related to the M2 phenotypic differentiation of macrophages. NEAT1 is highly expressed in exosomes derived from stem cells, which could be delivered to macrophages by exosomes, thereby promoting the M2-type polarization of macrophages, and the miR-125a/STAT3 axis may be involved in this process.

## Data Availability

The data presented in this study are not publicly available and are available on request from the corresponding author.

## Supplementary Materials

The following supporting information can be downloaded at https://www.mdpi.com/article/10.3390/cancers16142500/s1, Figure S1: The gating strategy; Figure S2: Relatively quantitative results of ELISA experiment of CCL2 levels in the culture medium of GL261 and GL261-GSC; Figure S3: Relatively quantitative results of qRT-PCR analysis of the expression NEAT1 in exosomes from U251/U251-GSC and A172/A172-GSC. GAPDH is used as a reference gene (*n* = 3); Table S1: Sequences of gene knockdown sites; Table S2: Sequences of primers used for qRT-PCR; Table S3: Primary antibodies used in the present study; Table S4: Sequences of predicted and mutant binding sites used for luciferase reporter assay.
