# Supplementary material for: Glioma-Stem-Cell-Derived Exosomes Remodeled Glioma-Associated Macrophage via NEAT1/miR-125a/STAT3 Pathway
Source: Cancers (Basel). 2024 Jul 9;16(14):2500. doi: 10.3390/cancers16142500 (PMC11274466; doi:10.3390/cancers16142500)
Supplement: Supplementary file 1 [file cancers-16-02500-s001.zip › Supplementary Material-1.pdf]

Supplementary Material

Figure S1: the gating strategy

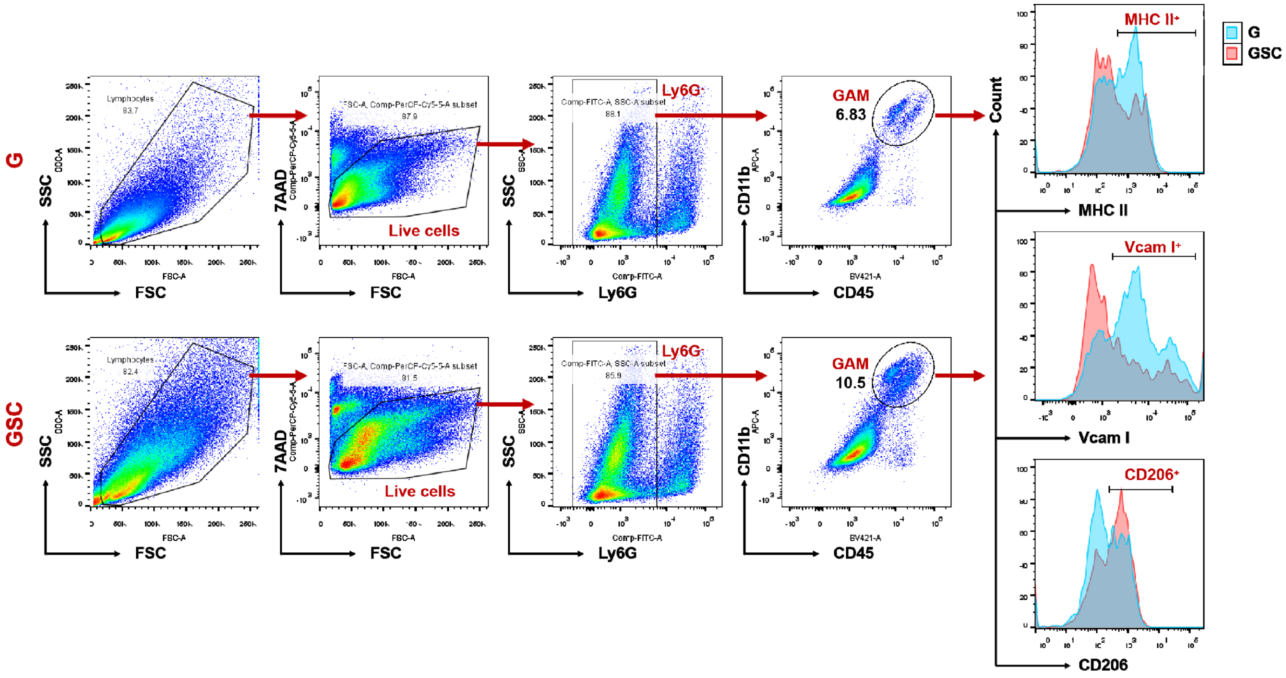

Figure S2: Relatively quantitative results of ELISA experiment of CCL2 levels in the culture medium of GL261 and GL261-GSC

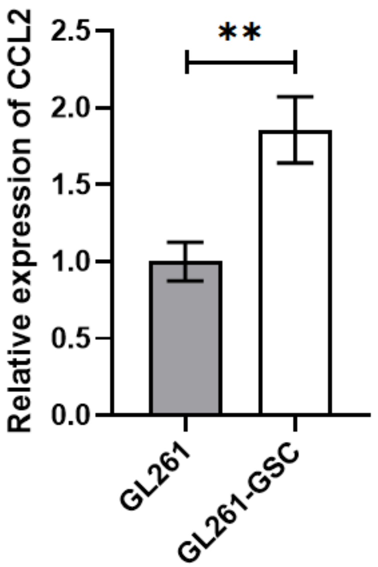

Figure S3: Relatively quantitative results of qRT-PCR analysis of the expression NEAT1 in exosomes from U251/U251-GSC and A172/A172-GSC. GAPDH is used as a reference gene (n=3)

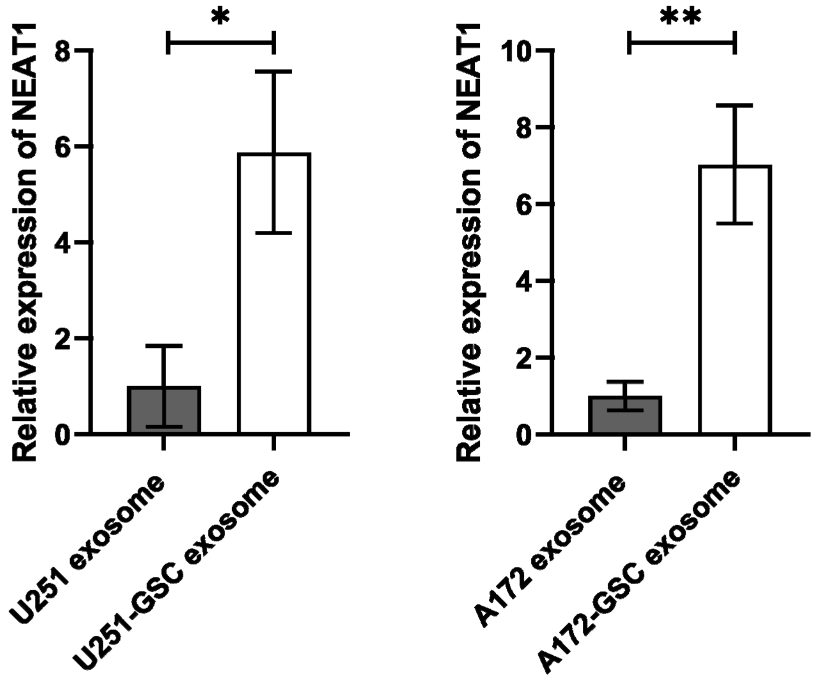

Table S1: Sequences of gene knockdown sites

Gene name	Sequence
Mus11891-siRab27a-1-F	GCUGUGUGGAAAUAAGAGUTT
Mus11891-siRab27a-1-R	ACUCUUAUUUCCACACAGCTT
Mus11891-siRab27a-2-F	GAGAGGUUUCGUAGCUUAATT
Mus11891-siRab27a-2-R	UUAAGCUACGAAACCUCUCTT
Mus11891-siRab27a-3-F	GCUUCUGUUCGACCUGACATT
Mus11891-siRab27a-3-R	UGUCAGGUCGAACAGAAGCTT
Neat1-Mus-1-F	GCUGUUAAACUGUCAGUCATT
Neat1-Mus-1-R	UGACUGACAGUUUAACAGCTT
Neat1-Mus-2-F	GGCGCAAGUUAGCCACAAATT
Neat1-Mus-2-R	UUUGUGGCUAACUUGCGCCTT

Neat1-Mus-3-F	GAACCAGCAGCUUUCAAGATT
Neat1-Mus-3-R	UCUUGAAAGCUGCUGGUUCTT

Table S2: Sequences of primers used for qRT-PCR

**Gene name**	**Sequence**
Arg-1(Mus)-F	TCTGCCAAAGACATCGTGTACAT
Arg-1(Mus)-R	CGACATCAAAGCTCAGGTGAATC
fizz1(Mus)-F	CCTCCACTGTAACGAAGACTCTC
fizz1(Mus)-R	CTCCCAAGATCCACAGGCAAAG
IL10(Mus)-F	CAGAGAAGCATGGCCCAGAAATC
IL10(Mus)-R	GCTCCACTGCCTTGCTCTTATTT
CD206(Mus)-F	TCCCTGTCTCTGTTCAGCTATTG
CD206(Mus)-R	CGTCTGAACTGAGATGGCACTTA
TGFβ(Mus)-F	GCGGACTACTATGCTAAAGAGGT
TGFβ(Mus)-R	GCTTCCCGAATGTCTGACGTATT
Rab27a(Mus)-F	TCGGATGGAGATTACGATTACCT
Rab27a(Mus)-R	TTTTCCCTGAAATCAATGCCCA
NEAT1(Mus)-F	AGGAGAAGCGGGGCTAAGTA
NEAT1(Mus)-R	TAGGACACTGCCCCCATGTA
miR-125a-5p-F	GCGTCCCTGAGACCCTTTAAC
miR-125a-5p-R	AGTGCAGGGTCCGAGGTATT
miR-125a-5p-RT	GTCGTATCCAGTGCAGGGTCCGAGGTATTCGCACTGGATACG ACTCACAG

CD133(Mus)-F	CTGGTGGGCTGCTTCTTTTGTATG
CD133(Mus)-R	CCGAGTCCTGGTCTGCTGGTTAG
SOX2(Mus)-F	GCGGAGTGGAAACTTTTGTCC
SOX2(Mus)-R	CGGGAAGCGTGTACTTATCCTT
Nestin(Mus)-F	CTGCTACCCTTGAGACACCTG
Nestin(Mus)-R	GGGCTCTGATCTCTGCATCTAC

Table S3: Primary antibodies used in the present study

**Antigens**	**Manufacturer**	**Catalogue numbers**	**Application**
TSG101	proteintech	28283-1-AP	1:1000 for WB
CD9	proteintech	20597-1-AP	1:1000 for WB
CD81	proteintech	66866-1-Ig	1:1000 for WB
Rab27a	abcam	ab55667	1:1000 for WB
STAT3	GeneTex	GTX104616	1:1000 for WB
Nestin	proteintech	19483-1-AP	1:50 for IHC
F4/80	abcam	ab111101	1:50 for IHC

Table S4: Sequences of predicted and mutant binding sites used for luciferase reporter assay

**Name**	**Sequence(5'- 3')**
STAT3 3'UTR WT 1107-1113 bp	CAGGCCCTCAGCAAAGCTCAGGGA

STAT3 3'UTR WT 1700-1707 bp	GGGGCTGGTGTTGTACCTCAGGGG
STAT3 3'UTR Mutant 1107-1113 bp	CAGGCCCTCAGCAAAGACTGCCCA
STAT3 3'UTR Mutant 1700-1707 bp	GGGGCTGGTGTTGTACACTGCCCG
miR-125a	AGUGUCCAAUUUCCCAGAGUCCCU
